# WHO Participates in Effectiveness Research? A Comparison of Effectiveness Trial Clinicians to National Survey Samples

**DOI:** 10.1007/s10488-022-01202-5

**Published:** 2022-06-14

**Authors:** Zabin S. Patel, Dominique Philips, Elizabeth Casline, Gregory A. Aarons, Colleen A. Maxwell, Golda S. Ginsburg, Jill Ehrenreich-May, Amanda Jensen-Doss

**Affiliations:** 1 Department of Psychology, University of Miami, Coral Gables, FL 33124 USA; 2 Department of Psychiatry, University of California San Diego, La Jolla, CA 92093 USA; 3 Department of Psychology, Temple University, Philadelphia, PA 19122 USA; 4 Department of Psychiatry, University of Connecticut, Farmington, CT 06030 USA

**Keywords:** Effectiveness trials, Community clinicians, Implementation science, Evidence-based practice, Measurement-based care

## Abstract

Findings from research participants in effectiveness treatment trials (i.e., randomized control trials conducted in community rather than research settings) are considered more generalizable than those from participants in efficacy trials. This is especially true for clinician participants, whose characteristics like attitudes towards evidence-based practices (EBPs) may impact treatment implementation and the generalizability of research findings from effectiveness studies. This study compared background characteristics, attitudes toward EBPs, and attitudes towards measurement-based care (MBC) among clinicians participating in a National Institute of Mental-Health (NIMH) funded effectiveness trial, the Community Study of Outcome Monitoring for Emotional Disorders in Teens (COMET), to clinician data from nationally representative U.S. survey samples. Results indicated COMET clinicians were significantly younger, less clinically experienced, and were more likely to have a training background in psychology versus other disciplines compared to national survey samples. After controlling for demographics and professional characteristics, COMET clinicians held more positive attitudes towards EBPs and MBC compared to national survey samples. Implications for implementation efforts are discussed.

An extensive literature describes the strengths of randomized control trials (RCTs), often considered the “gold-standard” for research evidence, for testing mental health interventions. A key advantage of RCTs is that the study design minimizes influence of confounders via random assignment so that causal relationships can be drawn (Essock et al., 2003). RCTs are commonly distinguished along a continuum of efficacy to effectiveness (Flay et al., 2005). Efficacy trials, which tend to be explanatory and precede effectiveness trials, test whether interventions work under controlled experimental conditions to maximize internal validity (Weisz et al., 1995). For example, an efficacy trial might test a mental health intervention delivered by highly trained and supervised staff or graduate students in a university-based research clinic. Effectiveness trials are RCTs carried out in routine, usual care conditions, like testing an intervention delivered by clinicians in community mental health clinics (CMHCs). Effectiveness trials are designed to balance emphasis on internal and external validity, or applicability of findings to wider settings and populations (Flay et al., 2005; Kessler et al., 2009). To address the research-to-practice gap in mental health services and advance public health, the National Institute for Mental Health (NIMH) has made effectiveness research a strong funding priority and there have been increasing calls for effectiveness research to assess intervention effectiveness in “real world” settings to increase generalizability and public health impact (NIMH, 2021).

An important consideration across the efficacy-effectiveness spectrum is whether the participants are representative of the larger population. An intervention shown to be efficacious and effective can only claim to be so for groups similar to the population from which participants were selected (Flay et al., 2005). There are several notable differences between the study samples in efficacy and effectiveness studies. Efficacy trials often tend to have stricter inclusion criteria and enroll more homogenous participants, in terms of demographic and clinical characteristics (Weisz et al., 1995). To support the generalizability of study findings, effectiveness trials typically have broader inclusion criteria and enroll more heterogenous samples (e.g., higher symptom severity, greater comorbidity) (Huebschmann et al., 2019; Singal et al., 2014; Tansella et al., 2006). Although it is generally assumed that effectiveness trial samples are more representative of the general population compared to efficacy trials, prior work has primarily focused on describing differences in patient samples (Persons & Silberschatz, 1998; Rengerink et al., 2017). To our knowledge, the question of sample representativeness in effectiveness research has not yet been examined in clinician samples.

In the Exploration, Preparation, Implementation and Sustainment (EPIS) framework, inner context factors, like organizational and individual clinician characteristics, are important determinants of implementation success (Aarons et al., 2011). Characterizing how clinicians that participate in effectiveness studies compare to the broader population of community mental health clinicians, for whom intervention delivery is intended, is important to inform the generalizability of treatment and implementation outcomes (e.g., the acceptability of evidence-based practices (EBPs) among practicing clinicians). For example, clinicians that participate in effectiveness trials may be more motivated to learn new EBPs, highly supervised, and more likely to participate in research opportunities compared to the broader population of community clinicians (McLeod et al., 2019). They may also have more specialized training consistent with EBP implementation and more favorable attitudes towards EBPs, which could translate to selection bias in research trials. These factors may potentially differentiate clinicians that participate in effectiveness trials from nationally representative clinician samples, which may limit our understanding of the research-to-practice gap in mental health services.

Comparing clinicians participating in effectiveness trials to nationally representative survey samples of mental health clinicians on attitudes towards EBPs is one way to address the question of representativeness. The Theory of Planned Behavior (Azjen, 1991), which predicts a person’s intentions to engage in a specific behavior, emphasizes the importance of attitudes in behavior change. Clinician attitudes towards EBPs have been widely studied as a clinician-level predictor of implementation, with mixed findings. Some studies have reported that clinicians with favorable attitudes towards EBPs are more likely to adopt them (Ashcraft et al., 2011; Lewis & Simons, 2011; Nakamura et al., 2011), while other studies have shown no association between attitudes and EBP use (Bearman et al., 2013; Higa-McMillan et al., 2015). Despite mixed findings, theoretical and empirical studies underscore the importance of attitudes in EBP adoption. As such, the representativeness of clinician attitudes towards EBPs warrants further investigation.

In this study, we compare demographic and professional characteristics, attitudes toward EBPs, and attitudes towards measurement-based care (MBC) among clinicians participating in a National Institute of Mental Health (NIMH) funded effectiveness trial (Community Study of Outcome Monitoring for Emotional Disorders in Teens; COMET; Jensen-Doss, Ehrenreich-May, et al., 2018; Jensen-Doss, Haimes, et al., 2018) to two national U.S. survey samples. National survey samples typically target the most prevalent providers of mental health services with the goal of obtaining representative samples of clinicians, and thus can serve as a useful reference for sample generalizability and cross-sample comparison. Survey samples were those used to establish the psychometric properties of three clinician attitudes measures: the Evidence-Based Practice Attitude Scale (EBPAS; Aarons, 2004; Aarons et al., 2010), the Attitudes Toward Standardized Assessment Scales-Monitoring and Feedback (ASA–MF; Jensen-Doss, Ehrenreich-May, et al., 2018; Jensen-Doss, Haimes, et al., 2018), and the Monitoring and Feedback Scale (MFA; Jensen-Doss, Ehrenreich-May, et al., 2018; Jensen-Doss, Haimes, et al., 2018). We hypothesized that COMET clinicians would have more positive attitudes towards EBPs and MBC compared to the national clinician samples.

## Methods

### Participants

176 clinicians were recruited to participate in the Community Outcome Monitoring for Emotional Disorders and Teens (COMET), a multi-site, randomized control trial testing the effectiveness of two EBPs for adolescent anxiety and depression in community mental health centers. COMET compared three conditions: (1) Treatment as usual; (2) Treatment as usual plus MBC (using the Youth Outcome Questionnaire; YOQ; Burlingame et al., 2005); and (3) Unified Protocol for Transdiagnostic Treatment of Emotional Disorders in Adolescents, a cognitive behavioral psychotherapy model (UP-A; Ehrenreich et al., 2008), plus MBC (Jensen-Doss, Ehrenreich-May, et al., 2018; Jensen-Doss, Haimes, et al., 2018). Clinicians were from 19 community mental health centers in the Northeastern and Southeastern United States and employed at least part-time. Each agency had an average of 9.26 (*SD* = 8.4; range 3–31) clinicians in the trial. COMET clinicians were on average 35.5 years old (*SD* = 10.1, range 23–65) and 85.8% (N = 151) female. 46.6% (N = 82) of the sample identified as Caucasian, 16.5% (N = 29) as African American, 32.9% (N = 57) as Hispanic, 0.6% (N = 57) as Asian American, and 3.4% (N = 6) as other race. Participant demographics are shown in Table 1.

**Table 1:** Comparisons between demographic and professional characteristics of Community Study of Outcome Monitoring for Emotional Disorders in Teens (COMET) and national U.S. survey samples

	COMET (N = 176)	EBPAS (N = 1089)	MFA & ASA (N = 504)
Age (years), *M *(*SD, range*)^a^			
	35.5 (10.1, 23–65)	38.22 (11.49, 21–73)	56.4 (11.69, 28–82)
		*t*(266.57) = 3.88, p < .01	*t*(351.68) = 22.47, p < .01
Female^b^			
	151 (85.8%)	816 (76%)	369 (73.9%)
		χ^2^(1) = 8.18, p = .004	χ^2^(1) = 9.66, p = .002
Ethnicity [N (%)]			
Caucasian	82 (46.6%)	689 (70.5%)	413 (89.6%)
African American	29 (16.5%)	146 (14.9%)	16 (3.5%)
Hispanic	57 (32.9%)	74 (7.6%)	16 (3.5%)
Asian American	1 (0.6%)	18 (1.8%)	6 (1.3%)
Other	6 (3.4%)	50 (5.1%)	10 (2.2%)
		χ^2^(4) = 96.56, p < .01	χ^2^(4) = 155.53, p < .01
Highest level of education [N (%)]			
Doctorate	7 (4.0%)	70 (7.0%)	75 (15.0%)
Master’s Degree	159 (90.3%)	677 (67.6%)	424 (85.0%)
Bachelor’s Degree	10 (5.7%)	229 (22.9%)	−
Less than Bachelor’s	–	25 (2.5%)	−
		χ^2^(3) = 39.14, p < .01	χ^2^(2) = 41.87, p < .01
Professional discipline [N (%)]^c^			
Social work	45 (25.6%)	407 (40.7%)	143 (28.4%)
Psychology	37 (21.0%)	320 (32.0%)	179 (35.5%)
Counseling	71 (40.3%)	–	−
Marriage & family	15 (8.5%)	–	182 (36.1%)
Education	2 (1.1%)	48 (4.8%)	−
Medicine/nursing	–	18 (1.8%)	−
Other	6 (3.4%)	206 (20.6%)	–
		χ^2^(1) = 54.33, p < .01	χ^2^(1) = 34.68, p < .01
Experience (years), *M *(*SD, range*)			
	4.25, (5.66, 0–25)	10.66 (8.51, 0–50)	22.2 (11.0, 2–55)
		*t*(309.27) = 12.45, p < .01	*t*(556.8) = 27.08, p < .01

Independent samples *t*-tests and χ^2^ tests compared the EBPAS and MFA & ASA–MF samples to the COMET sample

*EBPAS* Evidence-Based Practice Attitude Scale (Aarons et al., 2010), *MFA* Monitoring and Feedback Attitudes Scale (Jensen-Doss, Ehrenreich-May, et al., 2018; Jensen-Doss, Haimes, et al., 2018); *ASA–MF* Attitudes towards Standardized Assessment Scales–Monitoring and Feedback (Jensen-Doss, Ehrenreich-May, et al., 2018; Jensen-Doss, Haimes, et al., 2018)

^a^Participant age for the EBPAS sample was estimated using birth year and year of survey completion because date of survey completion was unavailable. As such, participant age used in this analysis (*M* = 38.84, *SD* = 11.4, range = 22–74) differs slightly from the participant age reported by Aarons et al. (2010) (*M* = 38.22, *SD* = 11.49, range = 21–73)

^b^1 COMET clinician identified as transgender (female to male), who was included with males given their gender selection

^c^Professional discipline was dichotomized to: 1 = Psychology (Psychology and Counseling) and 0 = Other (all other professional disciplines)

Aarons et al. (2010) participants (N = 1089) were recruited at 100 mental health clinics from 75 cities in 26 states; Jensen-Doss et al., participants (N = 504) were mental health clinicians recruited through three professional organizations (American Mental Health Counselors Association; American Association for Marriage and Family Therapy; National Association of Social Workers) that provided mailing lists of random, representative samples of their membership. Additional details on sample recruitment for the national survey samples can be found in Aarons et al. (2010) and Jensen-Doss, Ehrenreich-May, et al. (2018), Jensen-Doss, Haimes, et al. (2018).

### Measures

The *Clinician Background Questionnaire* is a brief questionnaire designed to capture clinician demographic and professional background information (theoretical orientation, degree, licensure status, years of professional experience, professional field).

The *Evidence-Based Practice Attitude Scale* (EBPAS; Aarons, 2004) is a 15-item instrument of a mental health clinician’s general attitudes towards EBP adoption. Clinicians rate on a 5-point scale the extent to which they agree with each item from 0 (“not at all”) to 4 (“a very great extent”). Responses are averaged into a total score and four subscales: Requirements (3 items, e.g., the extent to which a provider would adopt an EBP if it were required by an agency, supervisor, or state), Appeal (4 items, e.g., the extent to which a provider would adopt an EBP if it were intuitively appealing), Openness (4 items, e.g., the extent to which a provider is generally open to trying new intervention and would be willing to use more structured or manualized interventions), and Divergence (4 items, e.g., the extent to which the provider perceives EBPs are not clinically useful and less important than clinical experience). Higher scores indicate more positive attitudes towards EBPs, with the exception of the Divergence scale where lower scores indicate more positive attitudes. For the total scale score Divergence items are reverse scored prior to computing the mean subscale score. The EBPAS has demonstrated a stable factor structure that has been replicated across multiple studies, and has psychometric support for its use in clinician samples (Aarons, 2004; Aarons et al., 2007). The EBPAS has been translated into more than 18 languages and used in multiple settings including mental health, substance use disorder treatment, and other medical settings. For this study, the EBPAS validation sample was used as a comparison, which included a U.S. nationally representative sample of 1089 mental health service providers from 100 clinics across 26 states in the United States (see Aarons et al., 2010 for recruitment procedures; Table 1). Cronbach’s alpha for the subscales ranged from .67 to .91 (α_Total_ = .74) in the Aarons et al., 2010 sample, and from .61 to .92 in the current study (α_Requirements_ = .92; α_Appeal_ = .74; α_Openness_ = .81; α_Divergence_ = .61; α_Total_ = .85).

The *Monitoring and Feedback Attitudes Scale* (MFA; Jensen-Doss, Ehrenreich-May, et al., 2018; Jensen-Doss, Haimes, et al., 2018) is a 14-item measurement of clinician attitudes towards monitoring and feedback. Clinicians rate on a 5-point scale how much they agree with each item from 1 (“Strongly Disagree”) to 5 (“Strongly Agree”). Responses are averaged into two subscales: Benefits (10 items, e.g., the utility of monitoring and feedback for supervision and collaboration with clients) and Harm (4 items, e.g., whether negative feedback might harm the alliance or be misused by clinic administrators). The initial MFA validation sample consisted of 504 mental health providers recruited through the mailing lists of professional organizations (see Jensen-Doss, Ehrenreich-May, et al., 2018; Jensen-Doss, Haimes, et al., 2018 for recruitment procedures). Internal consistency for both scales were good (α_Benefit_ = .87; α_Harm_ = .87). In the current study, the MFA scales demonstrated good internal consistency (α_Benefit_ = .87; α_Harm_ = .78).

*Attitudes towards Standardized Assessment Scales*–*Monitoring and Feedback* (ASA–MF; Jensen-Doss, Ehrenreich-May, et al., 2018; Jensen-Doss, Haimes, et al., 2018) is an 18-item instrument that assesses clinician attitudes towards standardized instruments. The ASA–MF focuses specifically on standardized progress measures and their utility for clinical decision making. The ASA–MF provides participants with the definition of routine progress monitoring and standardized measures and asks them to indicate how much they agree with each statement on a scale of 1 (Strongly Disagree) to 5 (Strongly Agree). Responses are averaged across three subscales: Clinical Utility (8 items, e.g., MBC can provide useful clinical information, α = .85), Treatment Planning (8 items, e.g., MBC can be used to guide treatment decisions, α = .85), and Practicality (5 items, e.g., practical concerns like time or paperwork that may get in the way of using MBC, α = .81). The ASA–MF and MFA were developed using the same sample of 504 mental health providers. In the current study, internal consistency for the ASA–MF scales ranged from .77 to .82 (α_Clinical Utility_ = .77; α_Treatment Planning_ = .80; α_Practicality_ = .82).

### Procedures

CMHC clinicians were recruited from 19 clinics (6 in South Florida and 13 in New England). Interested clinicians were recruited via agency leaders, consented, and completed measures about their attitudes towards EBPs and using MBC prior to being randomized or trained. Once these measures were completed, clinicians were randomly assigned to one of the three treatment conditions. Clinicians received training and consultation in the UP-A and/or YOQ and treated COMET cases as part of their regular case load.

### Data Analysis

Rates of missing data were low (0–1.1%). Data were missing completely at random according to Little’s MCAR test (χ^2^ = 17.58, df = 12, p = .13) (Little, 1988), thus listwise deletion was used. Independent samples χ^2^ tests were used to compare COMET clinicians and the national survey samples on background characteristics; for χ^2^ calculation, some education and professional discipline categories were collapsed (see Table 1). Independent samples *t*-tests were used to compare samples on the EBPAS, MFA, and ASA–MF scale scores. The Benjamini & Hochberg false discovery rate (Benjamini & Hochberg, 1995) was used to correct for multiple comparisons (n = 20 analyses). Cohen’s d = .20, .50, and .80 were used to interpret effect sizes as small, medium, or large, respectively. Multiple regression models were used to compare the samples on scale scores after controlling for demographic and professional characteristics, given these variables may influence attitude scores. Analyses were conducted using R Version 4.0.3.

## Results

Comparisons between the COMET and the national survey samples are presented in Table 1. Demographically, compared to the national survey sample clinicians, the COMET sample clinicians were significantly younger (Aarons et al., 2010: *t*(266.57) = 3.88, p < .01; Jensen-Doss, Ehrenreich-May, et al., 2018; Jensen-Doss, Haimes, et al., 2018: *t*(351.68) = 22.47, p < .01), more female (Aarons et al., 2010: χ^2^(1) = 8.18, p = .004; Jensen-Doss, Ehrenreich-May, et al., 2018; Jensen-Doss, Haimes, et al., 2018: χ^2^(1) = 9.66, p = .002), and more ethnically diverse (Aarons et al., 2010: χ^2^(4) = 96.56, p < .01; Jensen-Doss, Ehrenreich-May, et al., 2018; Jensen-Doss, Haimes, et al., 2018: χ^2^(4) = 155.53, p < .01). Professionally, a higher proportion of COMET clinicians had a Master’s-level education (Aarons et al., 2010: χ^2^(3) = 39.14, p < .01; Jensen-Doss, Ehrenreich-May, et al., 2018; Jensen-Doss, Haimes, et al., 2018: χ^2^(2) = 41.87, p < .01) and a training background in psychology compared to other disciplines (Aarons et al., 2010: χ^2^(1) = 54.33, p < .01; Jensen-Doss, Ehrenreich-May, et al., 2018; Jensen-Doss, Haimes, et al., 2018: χ^2^(1) = 34.68, p < .01).

Sample scale scores are shown in Table 2. Compared to clinicians in the Aarons et al. (2010) sample, COMET clinicians had more positive EBP attitude scale scores, as measured by the EBPAS Total Score (*t*(227.55) = − 8.15, p < .0001, d = − .68 [95% CI − .85, − .52]). Analyses of the EBPAS subscales indicated COMET clinicians reported more positive EBP attitudes if it was required by their agency (*t*(233.7) = − 7.24, p < .0001, d = − .59 [95% CI − .75, −.43]) or it if was intuitively appealing (*t*(237.35) = − 3.18, p = .0016, d = − .25 [95% CI − .41, − 0.09]). COMET clinicians also reported greater openness to new interventions (*t*(237.35) = − 3.18, p = .0016, d = − .31 [95% CI − 0.47, − 0.15]) and were less likely to report perceiving interventions based in research as divergent from usual clinical practices (*t*(256.97) = 8.71, p < 0.0001, d = 0.63 [95% CI 0.47, 0.80]), when compared to the Aarons et al. (2010) sample.

**Table 2:** Scale score comparisons between Community Study of Outcome Monitoring for Emotional Disorders in Teens (COMET) and national U.S. survey samples

	National sample *M *(*SD*)	COMET *M *(*SD*)	Sample comparisons
EBPAS			
Requirements	2.41 (.99)	2.99 (.99)	*t*(233.7) = − 7.24, p < .0001, d = − .59 [95% CI − .75, − .43]
Appeal	2.91 (.68)	3.09 (.66)	*t*(237.35) = − 3.18, p = .0016, d = − .25 [95% CI − .41, − .09]
Openness	2.76 (.75)	2.98 (.67)	*t*(248.66) = − 4.08, p < .0001, d = − .31 [95% CI − .47, − .15]
Divergence	1.25 (.70)	0.81 (.59)	*t*(256.97) = 8.71, p < .0001, d = .63 [95% CI .47, .80]
Total score	2.73 (.49)	3.07 (.51)	*t*(227.55) = − 8.15, p < .0001, d = − .68 [95% CI − .85, − .52]
MFA			
Benefit	4.07 (.59)	4.35 (.42)	*t*(360.72) = − 6.98, p < .0001, d = − .58 [95% CI − .75, − .40]
Harm	2.45 (.69)	2.89 (.54)	*t*(394.82) = 12.99, p < .0001, d = 1.01 [95% CI .84, 1.2]
ASA–MF			
Clinical utility	2.98 (.64)	3.55 (.51)	*t*(383.1) = − 11.76, p < .0001, d = − .94 [95% CI − 1.12, − 0.76]
Treatment planning	3.35 (.70)	3.75 (.55)	*t*(394.33) = − 7.76, p < .0001, d = − .61 [95% CI − .79, − .43]
Practicality	3.13 (.73)	3.82 (.64)	*t*(354.98) = − 11.78, p < .0001, d = − .97 [95% CI − 1.15, − .79]

*EBPAS* Evidence-Based Practice Attitude Scale (Aarons et al., 2010), *MFA* Monitoring and Feedback Attitudes Scale (Jensen-Doss, Ehrenreich-May, et al., 2018; Jensen-Doss, Haimes, et al., 2018), *ASA–MF* Attitudes towards Standardized Assessment Scales–Monitoring and Feedback (Jensen-Doss, Ehrenreich-May, et al., 2018; Jensen-Doss, Haimes, et al., 2018)

Similarly, COMET clinicians reported more positive views of standardized progress measures than participants in the Jensen-Doss, Ehrenreich-May, et al. (2018), Jensen-Doss, Haimes, et al. (2018) study. On the MFA, they reported MBC as having more benefits (*t*(360.72) = − 6.98, p < .0001, d = − .58 [95% CI − .75,− .40]) and less harm (*t*(394.82) = 12.99, p < .0001. d = 1.01 [95% CI .84, 1.2]). COMET clinicians also reported higher scores on the ASA–MF indicating that standardized measures were more clinically useful (*t*(383.1) = − 11.76, p < .0001, d = − .94 [95% CI − 1.12, − .76]), beneficial for treatment planning (*t*(394.33) = − 7.76, p < .0001, d =− .61 [95% CI − .79, − .43]), and practical to use (*t*(354.98) = − 11.78, p < .0001, d = − .97 [95% CI − 1.15, − 0.79]) than did the Jensen-Doss, Ehrenreich-May, et al. (2018), Jensen-Doss, Haimes, et al. (2018) sample. All findings remained statistically significant (p < .05) after correcting for multiple comparisons using the Benjamini–Hochberg method (n = 20 analyses).

Given the significant differences in clinician background characteristics across study samples, multiple regression models were used to examine how scale scores compared after controlling for these variables. Results indicate that after controlling for demographic and professional characteristics, COMET clinicians continued to have more favorable attitudes towards EBPs (Table 3) and MBC (Table 4) compared to the national survey samples.

**Table 3:** Multiple regression models for the Evidence-Based Practice Attitude Scale (Aarons et al., 2010)

	Requirements			Appeal			Openness			Divergence			Total score		
Predictors	*β*	*CI*	*p*	*β*	*CI*	*p*	*β*	*CI*	*p*	*β*	*CI*	*p*	*β*	*CI*	*p*
(Intercept)	2.20	1.72 to 2.69	< 0.001	2.37	2.05 to 2.69	< 0.001	2.32	1.96 to 2.69	< 0.001	1.09	0.76 to 1.41	< 0.001	2.47	2.23 to 2.71	< 0.001
Sample: COMET	0.59	0.40 to 0.78	< 0.001	0.14	0.02 to 0.27	0.024	0.16	0.02 to 0.30	0.028	− 0.35	− 0.47 to − 0.22	< 0.001	0.29	0.20 to 0.39	< 0.001
Gender: female	0.26	0.11 to 0.40	0.001	0.11	0.02 to 0.21	0.022	− 0.01	− 0.12 to 0.10	0.884	− 0.08	− 0.18 to 0.02	0.122	0.10	0.03 to 0.17	0.008
Ethnicity: African American	− 0.09	− 0.27 to 0.08	0.294	− 0.36	− 0.47 to − 0.24	< 0.001	0.01	− 0.12 to 0.14	0.853	0.14	0.02 to 0.25	0.025	− 0.15	− 0.24 to − 0.06	0.001
Ethnicity: Hispanic	− 0.10	− 0.30 to 0.10	0.328	− 0.23	− 0.36 to − 0.10	0.001	0.02	− 0.13 to 0.17	0.791	− 0.04	− 0.17 to 0.10	0.586	− 0.07	− 0.17 to 0.03	0.173
Ethnicity: Asian American	− 0.04	− 0.49 to 0.41	0.862	0.08	− 0.22 to 0.38	0.600	0.02	− 0.32 to 0.36	0.917	0.10	− 0.20 to 0.40	0.506	− 0.01	− 0.23 to 0.21	0.939
Ethnicity: other	− 0.16	− 0.45 to 0.13	0.277	− 0.22	− 0.40 to− 0.03	0.026	0.04	− 0.18 to 0.25	0.746	0.16	− 0.03 to 0.35	0.095	− 0.11	− 0.25 to 0.03	0.113
Degree: Bachelor’s Degree	0.19	− 0.30 to 0.68	0.440	0.48	0.16 to 0.80	0.004	0.51	0.14 to 0.87	0.007	0.19	− 0.14 to 0.52	0.259	0.25	0.01 to 0.49	0.043
Degree: Master’s Degree	0.00	− 0.47 to 0.48	0.986	0.62	0.31 to 0.93	< 0.001	0.49	0.13 to 0.84	0.008	0.19	− 0.12 to 0.51	0.232	0.24	0.01 to 0.48	0.044
Degree: Doctorate	− 0.12	− 0.65 to 0.41	0.647	0.72	0.37 to 1.07	< 0.001	0.54	0.15 to 0.94	0.007	− 0.07	− 0.42 to 0.29	0.702	0.33	0.07 to 0.59	0.014
Professional Discipline	− 0.03	− 0.17 to 0.10	0.604	− 0.03	− 0.12 to 0.06	0.518	− 0.09	− 0.19 to 0.00	0.063	− 0.04	− 0.13 to 0.05	0.347	− 0.03	− 0.09 to 0.04	0.382
Age	0.01	− 0.00 to 0.01	0.133	0.00	− 0.00 to 0.01	0.615	0.00	− 0.00 to 0.01	0.325	− 0.00	− 0.01 to 0.00	0.709	0.00	− 0.00 to 0.01	0.202
Experience	− 0.02	− 0.03 to − 0.00	0.005	− 0.01	− 0.01 to 0.00	0.100	− 0.01	− 0.02 to − 0.00	0.002	0.01	0.01 to 0.02	< 0.001	− 0.01	− 0.02 to − 0.01	< 0.001
R^2^/R^2^ adjusted	0.081/0.070			0.087/0.076			0.032/0.021			0.093/0.082			0.105/0.095		

CI 95% Confidence Interval; Study sample was coded as a 2-level variable: 0 = national survey sample (Aarons et al., 2010), 1 = COMET sample; Gender was coded as a 2-level variable: 0 = male (1 COMET clinician identified as transgender (female to male), who was included with males given their gender selection); Ethnicity was coded as a 5-level variable, 0 = White; Highest professional degree was coded as a 4-level variable, 0 = Less than Bachelor’s; Professional discipline was coded as a 2-level variable: 0 = Other; 1 = Psychology and Counseling; Age and Experience scale is years

**Table 4:** Multiple regression models for the Monitoring and Feedback Attitudes Scale and Attitudes towards Standardized Assessment Scales–Monitoring and Feedback (Jensen-Doss, Ehrenreich-May, et al., 2018; Jensen-Doss, Haimes, et al., 2018)

	MFA benefit			MFA harm			ASA-MF Clinical utility			ASA–MF treatment planning			ASA–MF practicality		
Predictors	*β*	*CI*	*p*	*β*	*CI*	*p*	*β*	*CI*	*p*	*β*	*CI*	*p*	*β*	*CI*	*p*
(Intercept)	4.06	3.72 to 4.40	< 0.001	3.74	3.27 to 4.21	< 0.001	3.07	2.63 to 3.52	< 0.001	3.41	2.92 to 3.89	< 0.001	3.18	2.66 to 3.71	< 0.001
Sample: COMET	0.18	0.06 to 0.30	0.003	− 0.77	− 0.94 to − 0.61	< 0.001	0.40	0.24 to 0.56	< 0.001	0.25	0.08 to 0.42	0.005	0.56	0.37 to 0.74	< 0.001
Gender: female	− 0.09	− 0.18 to − 0.00	0.049	− 0.19	− 0.32 to − 0.07	0.003	− 0.17	− 0.29 to − 0.05	0.004	− 0.19	− 0.32 to − 0.07	0.003	− 0.16	− 0.30 to − 0.02	0.021
Ethnicity: African American	0.07	− 0.09 to 0.23	0.385	0.00	− 0.22 to 0.23	0.969	0.12	− 0.09 to 0.33	0.262	0.04	− 0.19 to 0.28	0.710	0.14	− 0.11 to 0.38	0.269
Ethnicity: Hispanic	0.14	0.01 to 0.28	0.040	0.03	− 0.15 to 0.22	0.731	0.07	− 0.11 to 0.25	0.433	− 0.06	− 0.25 to 0.14	0.582	0.02	− 0.19 to 0.23	0.873
Ethnicity: Asian American	0.10	− 0.27 to 0.48	0.584	0.19	− 0.33 to 0.71	0.466	0.34	− 0.15 to 0.83	0.168	0.15	− 0.38 to 0.68	0.579	0.14	− 0.44 to 0.71	0.645
Ethnicity: other	− 0.10	− 0.33 to 0.13	0.391	− 0.07	− 0.39 to 0.25	0.673	0.13	− 0.17 to 0.43	0.399	0.12	− 0.21 to 0.45	0.491	-0.10	− 0.45 to 0.26	0.595
Degree: Master’s Degree	0.07	− 0.26 to 0.40	0.663	− 0.02	− 0.48 to 0.43	0.921	0.06	− 0.36 to 0.49	0.768	0.14	− 0.33 to 0.60	0.562	0.08	− 0.43 to 0.59	0.754
Degree: Doctorate	0.17	− 0.18 to 0.52	0.342	− 0.08	− 0.57 to 0.41	0.751	− 0.08	− 0.54 to 0.38	0.736	0.10	− 0.39 to 0.60	0.681	0.02	− 0.51 to 0.56	0.928
Professional discipline	0.01	− 0.07 to 0.09	0.811	0.03	− 0.08 to 0.14	0.591	0.08	− 0.03 to 0.18	0.156	0.04	− 0.07 to 0.16	0.435	0.09	− 0.03 to 0.21	0.134
Age	− 0.00	− 0.01 to 0.00	0.306	− 0.01	− 0.02 to − 0.00	< 0.001	− 0.01	− 0.01 to − 0.00	0.032	− 0.01	− 0.01 to − 0.00	0.046	-0.01	− 0.01 to − 0.00	0.049
Experience	− 0.00	− 0.01 to 0.00	0.957	0.01	0.00 to 0.01	0.035	0.00	− 0.01 to 0.01	0.700	− 0.00	− 0.01 to 0.01	0.734	0.00	− 0.00 to 0.01	0.446
R^2^/R^2^ adjusted	0.082/0.065			0.215/0.200			0.173/0.157			0.089/0.072			0.170/0.154		

1 = COMET sample; Gender was coded as a 2-level variable: 0 = male (1 COMET clinician identified as transgender (female to male), who was included with males given their gender selection): 0 = male; Ethnicity was coded as a 5-level variable, 0 = White; Highest professional degree was coded as a 3-level variable, 0 = Bachelor’s; Professional discipline was coded as a 2-level variable: 0 = Other; 1 = Psychology and Counseling; Age and Experience scale is years

## Discussion

While it is generally accepted that samples from effectiveness trials are representative of the broader population, this question has not yet been tested in clinician samples. Understanding how effectiveness trial clinicians compare to the wider population of community mental health clinicians in practice is important for characterizing who participates in research studies and implementation efforts, contextualizing results, and comparing findings across study samples which may result in greater engagement with the research process and use of EBPs. To address this gap, we compared background characteristics and attitudes of clinicians participating in a NIMH effectiveness trial, COMET (Jensen-Doss, Ehrenreich-May, et al., 2018; Jensen-Doss, Haimes, et al., 2018), to national U.S. survey samples of community mental health clinicians (Aarons et al., 2010; Jensen-Doss, Ehrenreich-May, et al., 2018; Jensen-Doss, Haimes, et al., 2018). Consistent with hypotheses, results indicate COMET clinicians had more favorable attitudes towards EBPs and MBC compared to clinicians in the two survey samples after controlling for demographic and professional characteristics.

Results raise questions about how well effectiveness trials represent a test of evidence-based practices in community mental health. If the goal of effectiveness studies is to examine EBP delivery in routine practice settings with frontline providers, researchers should critically evaluate the background characteristics, attitudes, and experiences of clinician samples recruited in effectiveness studies. Our findings suggest that clinician samples used in effectiveness trials have highly positive attitudes towards EBPs compared to clinicians sampled from the community, which may translate to selection bias in recruitment and overestimation in the acceptability of EBPs. For example, given that COMET clinicians were recruited by their agency leaders to participate in the trial, these individuals may be more likely to value and engage in the research process (e.g., complete additional study measures, attend consultation calls), which may explain their favorable EBP attitudes. Indeed, recruitment procedures may influence the background characteristics and attitudes of clinicians selected to participate in COMET, and thus finding may not generalize to effectiveness trials that use other recruitment strategies (e.g., agency-wide EBP implementation). Future studies should examine whether variability in clinician attitudes towards EBPs and effectiveness trial recruitment procedures influence engagement in the research process.

Although it is possible that recruiting clinicians with more positive attitudes towards EBPs is an appropriate use of clinical research resources, future work should consider whether effectiveness trials are reaching clinicians with less favorable attitudes towards EBPs. Given that findings from effectiveness trials are often used to identify EBPs for widespread implementation, generalizing results from clinician samples with favorable attitudes may not appropriately predict implementation outcomes. It will remain important to critically examine how community clinicians and agencies are selected for effectiveness trials and develop strategies to engage clinicians with less positive attitudes in the research and EBP evaluation process.

Many studies describe a “voltage drop” in effectiveness when interventions are used in community settings (Chambers et al., 2013). It may be that clinicians who participate in research trials are more willing to adopt EBPs like MBC because of beliefs about the importance of evidence-informed practice. This could help explain the small effect sizes between the treatment as usual and EBP conditions observed in effectiveness trials, in that the usual care condition may be more “evidence-based” compared to routine clinical practice. Given clinician sample selection is an important consideration for both the usual care and intervention conditions, findings suggest that the usual care arm of an effectiveness trial may warrant closer examination (Löfholm et al., 2013). Describing the “voltage drop” in treatment effectiveness may also require further examining clinician-level characteristics, including demographic and professional variables (e.g., years of experience, theoretical orientation, productivity requirements, administrative responsibilities), which may influence EBP attitudes and consequently EBP use.

There are also study limitations. This was a retrospective study, a limited number of comparisons were made about clinician attitudes across the EBPAS, MFA, and ASA–MF scales. Additionally, given that nested data structures of the national survey samples were unknown, clustering was not accounted for in the analyses. The generalizability of effectiveness samples could be assessed across a wider range of practices and implementation characteristics including intervention fidelity, acceptability, and sustained use (Proctor et al., 2011). Future studies should also proactively investigate how clinicians who participate in effectiveness trials differ from those who choose not to (e.g., through a follow-up survey or qualitative interview), and identify predictors of early EBP adopters to help implementation efforts more efficiently allocate EBP training and intervention resources.

Second, although this study used national U.S. survey samples for comparison, survey samples are also research samples. It is possible that clinicians who participated in the surveys where the EBPAS, MFA, and ASA–MF were administered may have more favorable attitudes towards EBPs than those who did not participate. As such, it is possible that gaps between effectiveness samples and the general population of clinicians may be even more profound than those observed here. Third, this study did not assess or compare clinicians on professional or organizational factors, which may directly or indirectly influence EBP use and research participation. The importance of inner organizational context and outer system and policy context factors is well recognized in implementation frameworks like EPIS and should be further considered with respect to generalizability (Aarons et al., 2011).

Finally, given that the Aarons et al. (2010) sample was recruited over 10 years before the COMET sample and that both national survey samples had participants that were significantly older than the COMET sample, findings may be confounded by time. In other words, the positive attitudes that COMET clinicians hold may be reflective of attitudinal shifts as training and implementation efforts have emphasized EBPs over the past decade. Comparison of clinician background characteristics to the broader mental health workforce is also warranted. For example, the American Psychological Association estimates that in 2019, about 70% of psychologists were female, 83% were White, and 55% were between 31 and 50 years old (American Psychological Association, 2022). These demographics are generally reflective of the participants in the national survey samples used in this study, most of whom were masters-level providers. The COMET sample was more racially and ethnically inclusive than the U.S. clinician workforce, with 32.9% identifying as Hispanic, which raises questions about representativeness. Further study of how background characteristics moderate EBP attitudes over time is needed.

Overall, findings suggest that researchers should be cautious when concluding generalizability of findings to practicing clinicians from a single effectiveness trial. Use of innovative sampling methods, recruitment procedures, and study designs to encourage greater representation among clinicians and to evaluate EBP effectiveness in routine practice are important areas for further study. Studies can also examine how differences in attitudes about EBPs and the research process may influence clinician engagement in implementation as well as professional workplace advancement (e.g., more likely to finish training requirements, be promoted to clinical supervisor because of familiarity with EBPs, etc.). Gaining an understanding of factors that differentiate clinicians who participate in effectiveness research and those who do not can lend greater insight to the generalizability of study findings and the development of more targeted supports to increase EBP adoption among the broader population of community mental health clinicians.

## References

[CR1] Aarons GA (2004). Mental health provider attitudes toward adoption of evidence-based practice: The Evidence-based practice attitude scale (EBPAS). Mental Health Services Research Health Services Research.

[CR2] Aarons GA, Glisson C, Hoagwood K, Kelleher K, Landsverk J, Cafri G (2010). Psychometric properties and U.S. national norms of the evidence-based practice attitude scale (EBPAS). Psychological Assessment.

[CR3] Aarons GA, Hurlburt M, Horwitz SMC (2011). Advancing a conceptual model of evidence-based practice implementation in public service sectors. Administration and Policy in Mental Health and Mental Health Services Research.

[CR4] Aarons GA, McDonald EJ, Sheehan AK, Walrath-Greene CM (2007). Confirmatory factor analysis of the evidence-based practice attitude scale (EBPAS) in a geographically diverse sample of community mental health providers. Administration and Policy in Mental Health and Mental Health Services Research.

[CR5] Ashcraft RP, Foster SL, Lowery AE, Henggeler SW, Chapman JE, Rowland MD (2011). Measuring practitioner attitudes toward evidence-based treatments: A validation study. Journal of Child & Adolescent Substance Abuse.

[CR6] Ajzen I (1991). The theory of planned behavior. Organizational Behavior and Human Decision Processes.

[CR7] Bearman SK, Weisz JR, Chorpita BF, Hoagwood K, Ward A, Ugueto AM, Bernstein A, Research Network on Youth Mental Health (2013). More practice, less preach? The role of supervision processes and therapist characteristics in EBP implementation. Administration and Policy in Mental Health.

[CR8] Benjamini Y, Hochberg Y (1995). Controlling the false discovery rate: A practical and powerful approach to multiple testing. Journal of the Royal Statistical Society: Series B (Methodological).

[CR9] Burlingame GM, Cox JC, Wells MG, Lambert MJ, Latkowski M, Ferre R (2005). The administration and scoring manual of the Youth Outcome Questionnaire.

[CR10] Chambers DA, Glasgow RE, Stange KC (2013). The dynamic sustainability framework: Addressing the paradox of sustainment amid ongoing change. Implementation Science.

[CR11] *CWS data tool: Demographics of the U.S. psychology workforce*. American Psychological Association. Retrieved January 24, 2022, from https://www.apa.org/workforce/data-tools/demographics

[CR12] Ehrenreich, J. T., Buzzella, B. A., Trosper, S. E., Bennett, S. M., Wright, L. R., & Barlow, D. H. (2008). The Unified Protocol for treatment of emotional disorders in adolescents. *Unpublished Manuscript, Boston University*.

[CR13] Essock SM, Drake RE, Frank RG, McGuire TG (2003). Randomized controlled trials in evidence-based mental health care: Getting the right answer to the right question. Schizophrenia Bulletin.

[CR14] Flay BR, Biglan A, Boruch RF, Castro FG, Gottfredson D, Kellam S, Mościcki EK, Schinke S, Valentine JC, Ji P (2005). Standards of evidence: Criteria for efficacy, effectiveness and dissemination. Prevention Science.

[CR15] Higa-McMillan CK, Nakamura BJ, Morris A (2015). Predictors of use of evidence-based practices for children and adolescents in usual care. Administration and Policy in Mental Health.

[CR16] Huebschmann AG, Leavitt IM, Glasgow RE (2019). Making health research matter: A call to increase attention to external validity. Annual Review of Public Health.

[CR17] Jensen-Doss A, Ehrenreich-May J, Nanda MM, Maxwell CA, LoCurto J, Shaw AM, Souer H, Rosenfield D, Ginsburg GS (2018). Community study of outcome monitoring for emotional disorders in teens (COMET): A comparative effectiveness trial of a transdiagnostic treatment and a measurement feedback system. Contemporary Clinical Trials.

[CR18] Jensen-Doss A, Haimes EMB, Smith AM, Lyon AR, Lewis CC, Stanick CF, Hawley KM (2018). Monitoring treatment progress and providing feedback is viewed favorably but rarely used in practice. Administration and Policy in Mental Health and Mental Health Services Research.

[CR19] Kessler RC, Aguilar-Gaxiola S, Alonso J, Chatterji S, Lee S, Ormel J, Üstün TB, Wang PS (2009). The global burden of mental disorders: An update from the WHO world mental health (WMH) surveys. Epidemiologia e Psichiatria Sociale.

[CR20] Lewis CC, Simons AD (2011). A pilot study disseminating cognitive behavioral therapy for depression: Therapist factors and perceptions of barriers to implementation. Administration and Policy in Mental Health.

[CR21] Little RJA (1988). A test of missing completely at random for multivariate data with missing values. Journal of the American Statistical Association.

[CR22] Löfholm CA, Brännström L, Olsson M, Hansson K (2013). Treatment-as-usual in effectiveness studies: What is it and does it matter?. International Journal of Social Welfare.

[CR23] McLeod BD, Southam-Gerow MA, Jensen-Doss A, Hogue A, Kendall PC, Weisz JR (2019). Benchmarking treatment adherence and therapist competence in individual cognitive-behavioral treatment for youth anxiety disorders. Journal of Clinical Child and Adolescent Psychology.

[CR24] Nakamura BJ, Higa-McMillan CK, Okamura KH, Shimabukuro S (2011). Knowledge of and attitudes towards evidence-based practices in community child mental health practitioners. Administration and Policy in Mental Health.

[CR25] Oude Rengerink K, Kalkman S, Collier S, Ciaglia A, Worsley SD, Lightbourne A, Eckert L, Groenwold RHH, Grobbee DE, Irving EA (2017). Series: Pragmatic trials and real world evidence: Paper 3. Patient selection challenges and consequences. Journal of Clinical Epidemiology.

[CR26] Persons JB, Silberschatz G (1998). Are results of randomized controlled trials useful to psychotherapists?. Journal of Consulting and Clinical Psychology.

[CR27] Proctor E, Silmere H, Raghavan R, Hovmand P, Aarons G, Bunger A, Griffey R, Hensley M (2011). Outcomes for implementation research: Conceptual distinctions, measurement challenges, and research agenda. Administration and Policy in Mental Health and Mental Health Services Research.

[CR28] Singal AG, Higgins PDR, Waljee AK (2014). A primer on effectiveness and efficacy trials. Clinical and Translational Gastroenterology.

[CR29] Tansella M, Thornicroft G, Barbui C, Cipriani A, Saraceno B (2006). Seven criteria for improving effectiveness trials in psychiatry. Psychological Medicine.

[CR30] Weisz JR, Donenberg GR, Han SS, Weiss B (1995). Bridging the gap between laboratory and clinic in child and adolescent psychotherapy. Journal of Consulting and Clinical Psychology.

